# Supplementary material for: Genomic insights from a final Bronze Age community buried in a collective tumulus in an Urnfield settlement in Northeastern Iberia
Source: Commun Biol. 2025 Aug 28;8:1299. doi: 10.1038/s42003-025-08668-7 (PMC12394608; doi:10.1038/s42003-025-08668-7)
Supplement: Supplementary file 1 — Supplementary Information [file 42003_2025_8668_MOESM1_ESM.pdf]

Genomic Insights from a Final Bronze Age Community buried in a Collective Tumulus in an Urnfield Settlement in Northeastern Iberia

Authors: Marina Bretos Ezcurra[1,2], Adam B. Rohrlach[2,3], Luka Papac[2], José Ignacio Royo Guillén[4], Rodrigo Barquera[2], Fabiola Gómez Lecumberri[5], Rafael Laborda Lorente[1,6], Roberto Risch[7], Johannes Krause[2], Jesús V. Picazo Millán[1], Wolfgang Haak[2], Vanessa Villalba-Mouco[2,8].

[1] Institute for Research in Environmental Sciences of Aragon (IUCA), University of Zaragoza, Calle de Pedro Cerbuna 12, 50009 Zaragoza, Spain

[2] Department of Archaeogenetics, Max Planck Institute for Evolutionary Anthropology, Deutscher Platz 6, 04103 Leipzig, Germany

[3]School of Biological Sciences, University of Adelaide, Adelaide, South Australia, Australia

[4] Dirección General de Cultura y Patrimonio, Gobierno de Aragón, Avenida de Ranillas, 5 D., 50018 Zaragoza, Spain

[5] Independent researcher, Zaragoza, Spain.

[6] Paleoymás, Pol Empresarium C/retama 17, 24C. 50720 La Cartuja Baja-Zaragoza, Spain.

[7] Department of Prehistory, Universitat Autónoma de Barcelona, Barcelona, Spain.

[8] Institute of Evolutionary Biology, CSIC-Universitat Pompeu Fabra, Barcelona, Spain

Corresponding authors: Jesús V. Picazo Millán (jpicazo@unizar.es) , Wolfgang Haak (wolfgang_haak@eva.mpg.de), Vanessa Villalba-Mouco (vanessa_villalba@eva.mpg.de)

Author contribution: This study was designed by W.H. and V.V.-M. Sample preparations and laboratory work was conducted by R.B, J.I.R.G., R.L. and V.V-M., and subsequent analyses were performed and discussed by M.B., V.V.-M., A.B.R. L.P. and W.H. The integration of the archaeological data was performed by M.B.,

J.V.P. and R.R. The original manuscript was prepared by M.B., V.V.-M., W.H. and A.B.R. and reviewed by all co-authors.

Competing Interest Statement: The authors declare no competing interest

Keywords: Ancient DNA, biological relatedness, kinship, ancient population genomics, cremations, Indo-European languages, Late Prehistory.

Supplementary Information

Table of contents:

- SI 1 - Archaeological information of the studied sites
- SI 2 - Relatedness between LCA007.A and LCA010.AB using BREADR and KIN
- SI 3 - Population genomic analysis
    - 3.1 Evaluation of PCA shifts depending on the data treatment.
    - 3.2 Steppe ancestry quantification over BA and IA in Northeastern Iberia
    - 3.3 Steppe ancestry increase through f_4-*statistics* of the form *f4(Mbuti, test; Russia_Samara_EBA_Yamnaya, Turkey_N)*
    - 3.4 Rotating qpAdm models confirm that the southeastern Iberian group is a better proxy than the local northeastern one
- SI 4 - Archaeological sampling permit issued by "Gobierno de Aragón"
- References

Other Supplementary Material:

- **Supplementary Data 1. Descriptive summaries of genomic results:**
  1.1 Archaeological context and main genomic data

  1.2 1240k EAGER data

  1.3 Mito-capture EAGER data

  1.4 Mitochondrial haplogroup assignment

  1.5 Y-chromosome capture EAGER data

  1.6 Sex Determination analysis

  1.7 READ-KIN-BREADR analysis

1.8 Pairwise mismatch rate (PWMR) analysis

1.9 IBD results with at least one IBD segment >20 centiMorgans using ancIBD

1.10 Runs of homozygosity using HapROH

- **Supplementary Data 2. Details of genomic data analyses:**

  2.1 Group labels and comparative ancient dataset used in this study

  2.2 qpAdm results with the distal model

  2.3 Kruskall Wallis Test of Steppe proportion in different groups measured by qpAdm distal model and PC2 coordinates

  2.4 f4 statistics

  2.5 qpAdm Autochthonous

  2.6 qpAdm Local + C.European or Mediterranean

  2.7 qpAdm SE Iberian + C.European

  2.8 qpAdm WesternMed + C. European

SI 1 - Archaeological information of the studied sites

<u>Los Castellets II (Tumulus 2)</u>

Archaeological collaborators: José Ignacio Royo Guillén, Fabiola Lecumberri

The site is described in [1–8]. The archaeological complex of Los Castellets is located in the municipality of Mequinenza, near the confluence between the Segre and the Ebro rivers at the Northeastern Iberian Peninsula. The site extends over two rocky spurs, separated by a deep ravine.

The site was discovered in January 1976 by the Archaeological Group of Mequinenza (G.A.M.). They initiated unofficial excavations uncovering two houses from the settlement, as well as eleven tumulus burials. The discovery of ceramics with cylindrical button appendixes handles of the type “ad ascia” suggested the chronology of Final Bronze Age (FBA) [9]. The initial official excavation campaign was conducted in 1983 ([1–8]), followed by a total of ten additional fieldwork campaigns since then. Throughout this period, several studies have been published addressing various aspects of the site, including its chronology, tumulus typology, funeral rituals, and artifacts recovered [1–8]. All excavation efforts have been exclusively focused on the archaeological documentation of the necropolis Los Castellets II, and the settlement area remains unstudied for now.

The settlement area is located on the hill situated farthest to the east and presents material culture associated with several chronological periods from the Middle Bronze Age (MBA) to the Iberian culture (Late Iron Age, LIA), including characteristic items from the Urnifield culture and the Iron Age. The strategic position of this settlement is evident, as it controls the passage through the valley, and is also very close to the confluence of the Cinca and Segre rivers with the Ebro, which were excellent natural routes in the past. It is delimited in the north by high mountains, at the extreme southwest by a cliff over the Ebro River, completely inaccessible, and by two deep ravines to its left and right. Therefore, access to it is achieved through steep slopes, making it very challenging. At the northeastern end of the spur lies the wall that completely encloses the spur, thus defending the site at its weakest point. The settlement developed around the wall, with rectangular dwellings directly built on the

limestone quarry. In some areas, remnants of walls, about 50 cm in height, constructed with stone masonry, are still preserved.

Next to the settlement there is an extensive incineration mound necropolis assigned to the FBA/IA Urnfield Culture, named Los Castellets I, that has not been excavated yet. The area dedicated to the tumuli of incineration is extensive. These tumuli are more or less circular, ranging in diameter from 2m to 3m. The tumulus is constructed with limestone slabs inserted into the ground.

The western spur is called Los Castellets II and is largely occupied by a mixed inhumation and cremation mound necropolis that spans a chronology from the FBA (~1200 BCE) to the Late Iron Age (~500 BCE). Funerary steles, worked and unworked, and funerary cippes of anthropomorphic, cylindrical and indeterminate type have been found. Regarding the radiocarbon chronology of Castellets II, although dates are relatively scarce, the archaeologists state with absolute certainty the coexistence of the two funerary rituals (Supplementary Figure 1). At Castellets I, the oldest ceramic materials can confidently be dated to the 11th–10th centuries BCE. This necropolis displays tumuli and cist typologies identical to those at Castellets II, although no inhumation graves have been documented, only cremation burials. Between the 11th and 9th centuries BCE, there is sufficient evidence to confirm the coexistence of both funerary rituals. However, inhumations appear to have been reserved for certain social or familial groups, as these graves are located in the most prominent areas of the necropolis and are consistently surrounded by cremation tumuli.

The tumulus studied in the present article, Tumulus 2, as described in the main text, was selected for its significance in comparison to the other excavated tumuli (over 50 in total). Its size, location, and the number of individuals buried within it render it exceptional in its context, as there are no other collective tumuli of such magnitude. There were several infant individuals interred in the tumulus, but no samples could be taken from them. The precise description appears in the main text.

Apart from the Tumulus 2, there are other tumuli of interest with particular traits. For example, the tumulus 14 consists of a circular tumulus mound, reaching almost 1 meter in height and with a diameter of approximately 5 meters. At the centre of this mound stands an elongated stele serving as a marker. Right at this central point of the tumulus, the burial chamber is placed, rectangular in shape, measuring about 1.60 meters in length by 1.20 meters in width, with a small indentation on the west

side corresponding to the head end. The chamber is constructed with two to three rows of large limestone orthostats, forming walls slightly sloping inwards. Inside the chamber were found the remains of a simple burial, possibly in a fetal position, with the head resting on a slab at the head end. Accompanying the human remains was a funerary assemblage including bronze bracelets and rings, as well as offering vessels with limestone lids. This assemblage stands out as the richest in the necropolis, which is generally characterised by its austerity and scarcity of metallic artifacts. Charcoal from the burial ground was radiocarbon dated, offering a date of 1090 cal. BCE [5,10–12]. Another burial with the same characteristics was found, Tumulus 28 [10,11]. The funerary structure consists of a circular tumulus mound, with a diameter of approximately 4 meters. The chamber of the burial is constructed with 4 large limestone orthostats. The body was placed in a fetal position on his right side, facing the West. This person, identified as a male individual between 38 and 45 years old, had a stone beneath his head, in the form of a pillow to rest it.

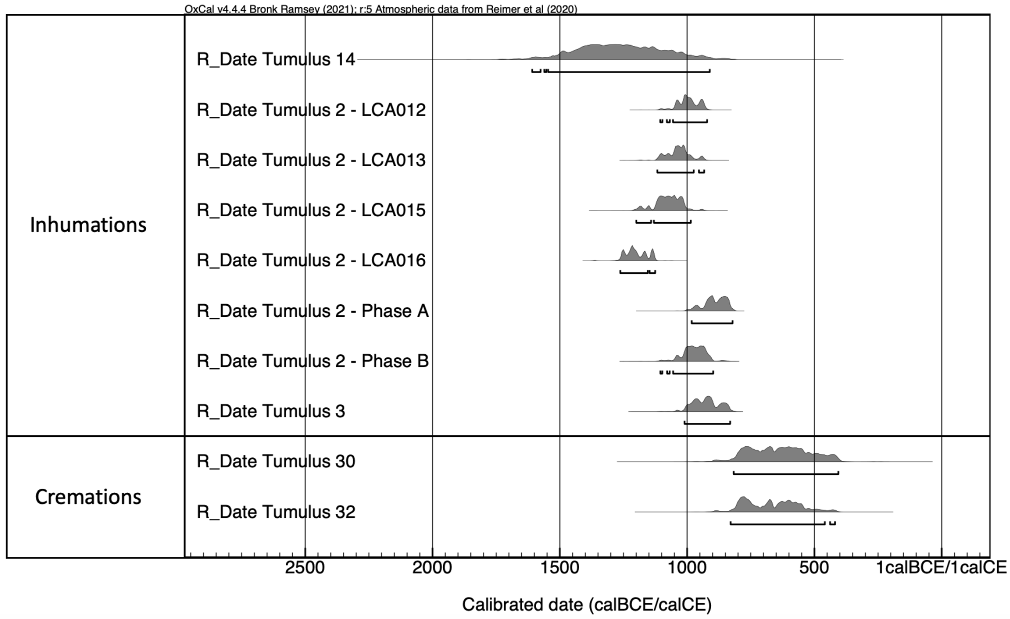

Supplementary Figure 1. Set of calibrated dates (2sigma) performed in bone and charcoal samples from Los Castellets II, from both inhumations and cremations. All inhumation datations were performed in bone, while datations in cremations were performed in charcoal.

Los Piojos Cave

<u>Archaeological collaborators</u>: Rafael Laborda Lorente, Vanessa Villalba-Mouco

Los Piojos cave is located in the northern foothills of the Iberian System, in the municipal area of Ricla (Zaragoza, Spain). The cave develops in Jurassic limestones, and has a length of 34 m., with a relative height difference of 4 m. Its entrance opens to the south, with a vertical duct of 2 m corresponding to the drainage of a sinkhole developed on a stratification plane. This duct reaches the base of a cone that occupies most of the cave. At the base of it, two galleries open, called "Gatera" and "Zona Norte" [13]. The "Gatera" area is characterised by the presence of a shelf filled with hundreds of limestone blocks. In the "Zona Norte," there is an accumulation of sediment.

During the field work carried out in 2016 by Consolidated research group “Primeros Pobladores del Valle del Ebro” and “Centro de Espeleología de Aragón” and published in 2017 [14], archaeological remains were recovered in both areas of the cave, "Gatera" and "Zona Norte." The human remains analysed in this present work correspond to those of an individual represented only by a mandible found at a superficial level from “Zona Norte”. Its radiocarbon dating yielded an age of 760-431 cal BCE (2462± 22 BP), encompassing a wide range during the Iron Age.

The rest of the individuals found in this cavity area belong to a closed sepulchral ensemble represented by human remains and funerary grave goods dating back to the late Neolithic/Chalcolithic period with a direct dating of 3089-2907 cal BCE, (4372±30 BP). Their analysis is published in [14] although they are not included in the genetic study of this present work.

SI 2 - Relatedness between LCA007.A and LCA010.AB using BREADR and KIN

We dedicate an additional supplementary analysis to determine the most likely degree of relatedness between sample LCA007.A and sample LCA010.AB. Sample LCA007.A is a lower left third molar, and sample LCA010.AB is a merging of data obtained from LCA010.A (left petrous bone) and LCA010.B (upper left third molar) coming from the same skull. We used BREADR and KIN to estimate the degree of

relatedness between LCA007.A and LCA010.AB. BREADR method reported a 1st-degree relationship as the most likely scenario, although being identical individuals or twins had a fairly high posterior probability of 0.4, leaving some doubt whether LCA010 and LCA007 might actually be the same individual (Supplementary Figure 2 A,B). However, the software KIN reported a 1st-degree parent-offspring relationship as the most likely, both for the combination of LCA010.A and LCA007.A and for LCA010.AB and LCA007.A. Yet, the most likely degree of relatedness for the pair LCA010.B and LCA007.A reported again as identical individuals or twins although overlapping number of SNPs are lower in this pair combination (Supplementary Data 1.7).

Additionally, we estimated contamination with ANGSD since LCA010.AB and LCA007.A are both genetic males. First, we saw that LCA010.AB reports contamination levels of 2.8% and merging of LCA010.AB and LCA007.A did not increase contamination (2.5%). The absence of an increased contamination expected if merging two different individuals was not observed. But this result can be explained because of the low SNP coverage of the individual LCA007.A who only increased the SNPs covered on the X chromosome twice required to estimate contamination in nine sites.

Finally, based on all this data together, we considered LCA010.AB and LCA007.A 1st-degree as the most likely scenario, but due to the SNP coverage of LCA007.A, the results should be taken with caution.

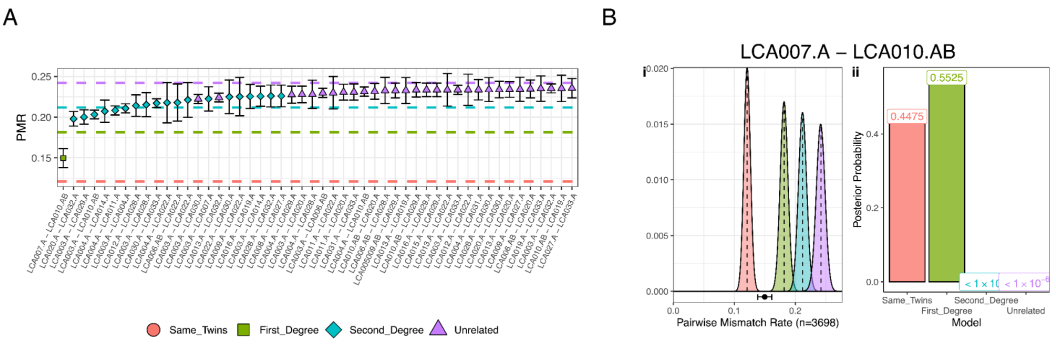

Supplementary Figure 2. BREADR results of individuals from Los Castellets II. A) Site-wise results of relatedness with 95% confidence intervals and the level of genetic relatedness with the highest posterior probability indicated by colour and shape, B) Genetic relatedness results for the pair LCA007.A and LCA010.AB. (i) The distribution of the expected pairwise mismatch rates values for each degree of relatedness, given the number of overlapping SNPs, with the observed pairwise mismatch rates (and 95% confidence interval) displayed below, and (ii) the posterior probabilities of each degree of relatedness for the pair.

SI 3 - Population genomic analysis

3.1 Evaluation of PCA shifts depending on the data treatment.

When we perform our PCA, we see that the individuals from Castellets II are somewhat displaced upwards and to the left, which would indicate a slightly higher level of steppe ancestry than the preceding EBA populations from the Northeastern Iberian Peninsula. However, it is known that the type of treatment given to the BAM files (number of bases trimmed depending on the damage at the ends of the reads) and the type of genotyping used can cause small displacements in PCA space. Since this is the first time that data produced by single-stranded non-UDG libraries has been published for the Iberian Peninsula, we wanted to investigate whether the position of the individuals in PCA space might be biased due to data treatment rather than their genetic composition. In order to assess which genotyping techniques performed better in PCA, we ran three PCA analyses with different genotyping modes (Supplementary Figure 3):

1. Samples were genotyped with --pseudohaploid --singlestrandedmode.
2. Samples were genotyped with --pseudohaploid and standard mode, but the last 10bp were trimmed from reads in the BAM files.
3. Samples genotyped with --pseudohaploid and standard mode, but without trimming the reads in the BAM files.

After this evaluation, we clearly observed that method one produced the most consistent positions in PCA for the new individuals from our study, when compared to the published individuals, and we thus chose this method for all our analyses.

Additionally, in SI 3.3, we see that the ancestry proportions estimated by qpAdm matches what is expected for the position of the individuals in PCA space (see below).

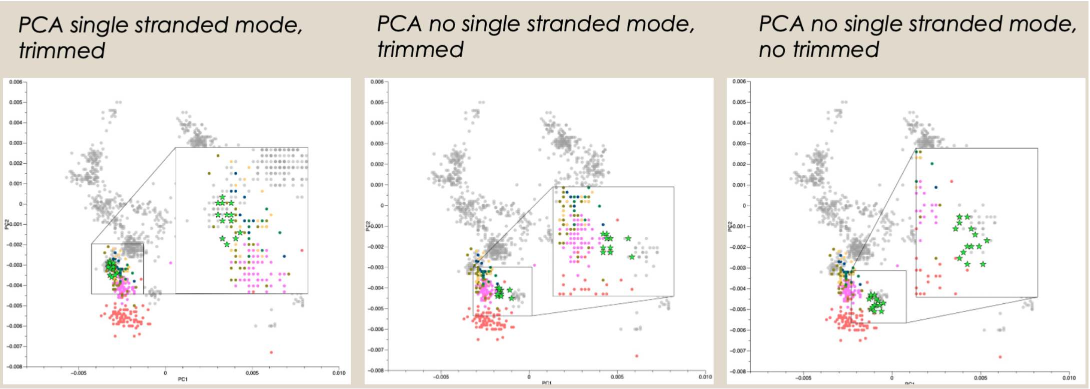

Supplementary Figure 3: West Eurasian PCA including our Los Castellets II individuals whose data was treated in three different ways. From left to right: Samples genotyped with --pseudohaploid --singlestrandedmode; samples genotyped with --pseudohaploid and standard mode trimming the last 10bp of the reads in the BAM files; samples genotyped with --pseudohaploid and standard mode without trimming the reads in the BAM files

3.2 Steppe ancestry quantification over time in Northeastern Iberia

In this study we evaluated the relative proportion of steppe-related ancestry using different approaches. The most general approach is through the position each individual takes on the second principal component (PC2), since it has been shown that there is a positive correlation between PC2 values and the proportion of steppe ancestry in Iberian BA (Villalba-Mouco et al. 2021). The second approach involves fitting a distal qpAdm model following Patterson et al. (2022) (see Fig. 2B). Both approaches show a coefficient of determination (r^2) of 0.784 (Supplementary Figure 4). The results obtained show a positive but not significant trend over the time using both methods as a proxy for the steppe ancestry quantification: PC2 coordinate and qpAdm Steppe proportion (p-value <0.05). However, a Pairwise Wilcoxon Rank Sum test shows only significant differences (p-values <0.05) between BA and IA groups (Supplementary Figure 4 A, B, C, Supplementary Data 2.3). This suggests that the increase in steppe-related ancestry already began in the FBA but increased further in a subtle and prolonged manner until the LIA.

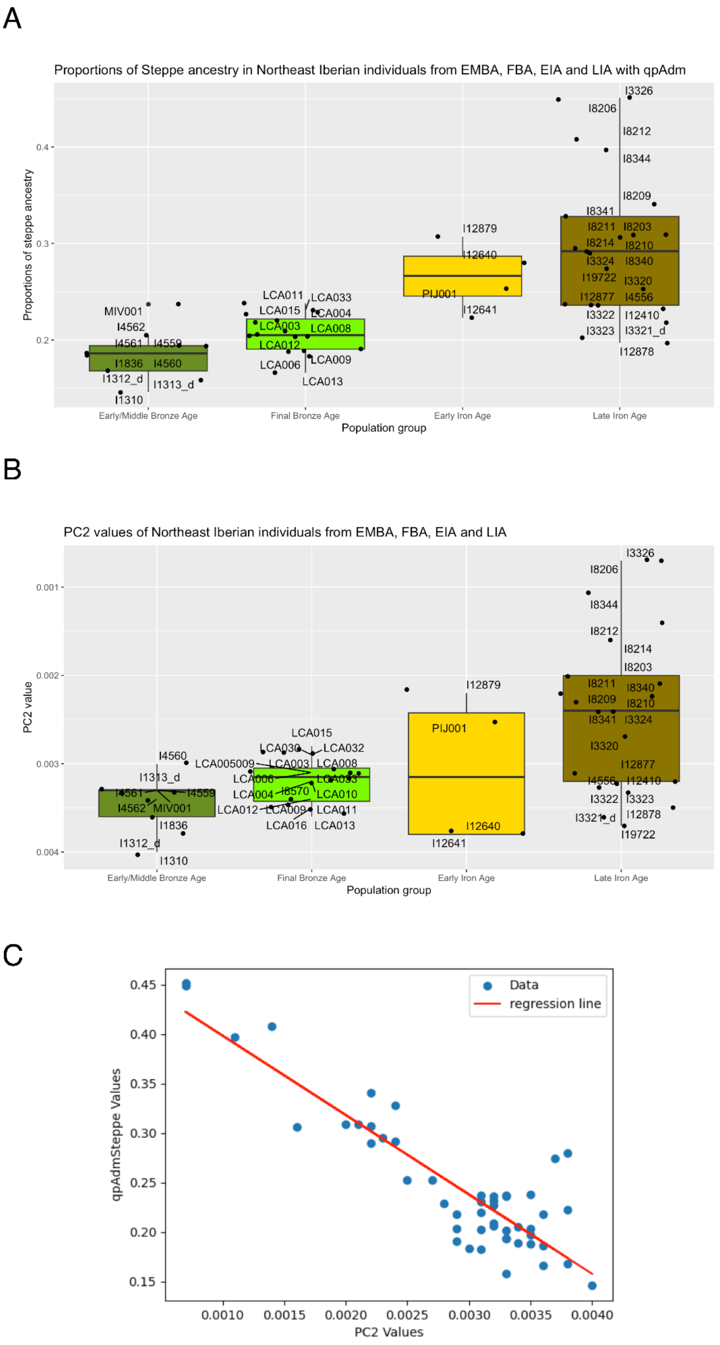

Supplementary Figure 4. A) Comparison of the proportion of steppe ancestry in different groups (EBA, FBA, EIA and LIA) estimated using a distal qpAdm model. A Kruskal-Wallis test indicated significant differences (p-value <0.05). Post-hoc Pairwise Wilcoxon Rank Sum tests indicate no significant differences (p-value >0.05) between EBA vs LBA, LBA vs EIA or EIA vs LIA, but significant for the rest of the comparisons (p-value < 0.05) (Supplementary Data 2.3). B) Comparison of Steppe proportion in different groups (EBA, LBA, EIA and LIA) measured by PC2 coordinate. A Kruskall-Wallis test showed no significant differences (p-values >0.05) between EBA vs LBA, LBA vs EIA or EIA vs LIA, but significant in the rest of the comparisons (p-value 0.05) (Supplementary Data 2.3). C) Correlation between PC2 values and qpAdm proportions. Correlation coefficient (R): -0.886, coefficient of determination (R^2): 0.784, p-value: 1.338e-17

3.3 Steppe ancestry increase through *f_4-statistics* of the form f4(Mbuti, test; Russia_Samara_EBA_Yamnaya, Turkey_N)

Finally, we also estimated the increase of steppe ancestry over the time using the f4-statistics of the form *f4(Mbuti, test; Russia_Samara_EBA_Yamnaya, Turkey_N),* where more negative values are indicative of higher levels of Steppe ancestry (Supplementary Figure 5).

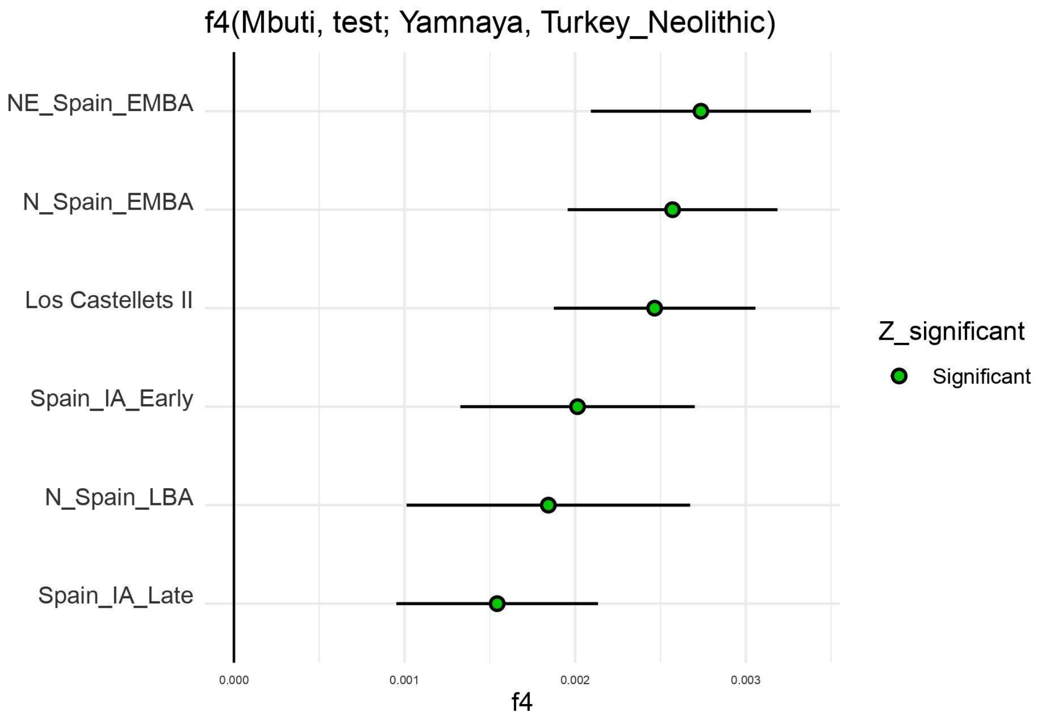

Supplementary Figure 5. Gradient of steppe-related ancestry in northeast Iberian individuals. F4-statistics showing the presence of steppe-related ancestry in all the reported NE groups, confirming a gradient from EMBA (lower steppe-related ancestry) to IA (higher steppe-related ancestry).

3.4 Rotating qpAdm models confirm that the southeastern Iberian group is a better proxy than the local northeastern one

In order to test if the genetic ancestry represented by the Southeastern_Iberia_EMBA is a better proxy for the Iberian ancestry found at Los Castellets II than the local EMBA, we performed a rotating qpAdm model using Southeastern_Iberia_EMBA + Central Europe_BA as sources adding Local EMBA (NE_Iberia_EMBA or N_Iberia_EMBA) in the outgroup set. This analysis yields p-values of the same order of magnitude (from 0.03 to 0.09), indicating that Southeastern Iberia EMBA is a better

proxy for the “local” ancestry found in Los Castellets II than the preceding NE_Iberia_EMBA or N_Iberia_EMBA.
However, when placing Southeastern_Iberia_EMBA in the outgroup set, and using either NE_Iberia_EMBA or N_Iberia_EMBA as a source in combination with Central Europe_BA, the model is rejected (p-values from 5.79×10-06 to 6.43×10-05) (Supplementary Data 2.7).

SI 4 - Archaeological sampling permit issued by "Gobierno de Aragón"

GOBIERNO DE ARAGON
Departamento de Educación, Cultura y Deporte

RESOLUCIÓN DE LA DIRECCIÓN GENERAL DE PATRIMONIO CULTURAL PARA LA PARA LA EXTRACCIÓN DE MUESTRAS ÓSEAS DE LOS RESTOS HUMANOS DE LA NECRÓPOLIS DE LOS CASTELLETS II, EN EL T.M DE MEQUINENZA (ZARAGOZA) PARA ESTUDIO GENÉTICO

Vista la solicitud para la realización de la extracción de muestras óseas de los restos humanos de la necrópolis de los Castellets II, depositados en los almacenes de la antigua Universidad Laboral de Zaragoza, sita en el Polígono Malpica, para la realización de un estudio genómico integral de los individuos no incinerados, en especial de los recuperados en el túmulo nº 2, formulada con fecha 04 de julio de 2019 por D. José Ignacio Royo Guillen y Dª Vanesa Villaba Mouco y habida cuenta de que la documentación que acompaña a la citada solicitud se ajusta a lo dispuesto en el Decreto 6/1990, de 23 de enero, de la Diputación General de Aragón, por el que se aprueba el régimen de autorizaciones para la realización de actividades arqueológicas y paleontológicas en la Comunidad Autónoma de Aragón, y en la ley 3/1999, de 10 de marzo, de Patrimonio Cultural Aragonés, la Dirección General de Patrimonio Cultural,

AUTORIZA A LA paleoantropóloga Dª Vanessa Villaba Mouco a la realización de dicha actuación, financiada por*Department of Archaegenetics* del *Max Planck Institute for the Science of Human History*.

LA ACTUACIÓN CONSISTIRÁ en la extracción, bajo la supervisión de la técnico especialista de la Dirección General Dª Belén Gimeno Martínez, de muestras óseas de los restos humanos de la necrópolis de los Castellets II, depositados en los almacenes de la antigua Universidad Laboral de Zaragoza, sita en el Polígono Malpica, para la realización de un estudio genómico integral de los individuos no incinerados, en especial de los recuperados en el túmulo nº 2. El estudio se llevará a cabo en el *Department of Archaegenetics* del *Max Planck Institute for the Science of Human History*.

De cada individuo se solicita la extracción de una pieza dental y/o la porción petrosa del hueso temporal (albergada en la parte interna del cráneo). Las piezas dentales se cortarán por la unión de la raíz y la corona para perforar la cavidad pulpar (vaciado interior). La técnica no afecta a la morfología dental y las coronas se pueden volver a adjuntar en la cavidad dental en el caso que sea necesario. En el caso de las porciones petrosas, éstas se perforarán con un taladro desde el exterior generando un agujero de 4 mm de diámetro. Dependiendo de los resultados genéticos, es posible que se tome alguna muestra para datación por Carbono 14 de las mismas porciones petrosas y/o raíces dentales. Los restos

óseos utilizados, se devolverán a su lugar de depósito, una vez concluido el estudio genético.

Se realizará un inventario de las muestras extraídas y un aparato gráfico del proceso, así como un acta de entrega y recepción de los mismos.

Los restos óseos utilizados, se devolverán a su lugar de depósito, una vez concluido el estudio genético.

Zaragoza 05 de julio de 2019

Fdo. D. Ignacio Escuín Borao

DIRECTOR GENERAL DE PATRIMONIO CULTURAL

GOBIERNO DE ARAGÓN
DEPARTAMENTO DE EDUCACIÓN, CULTURA Y DEPORTE
DIRECCIÓN GENERAL DE PATRIMONIO CULTURAL

333

References

1. Royo Guillén, J. I. & Ferreruela Gonzalvo, A. Noticia preliminar sobre la necrópolis de inhumacion e incineración de Los Castellets (Mequinenza, Zaragoza). *Boletín del Museo de Zaragoza* (1983).
2. Royo Guillén, J. El yacimiento de Los Castellets y su necrópolis tumular de inhumación e incineración. (Mequinenza, Zaragoza). *Arqueología Aragonesa* **1984**, 47–53 (1986).
3. Royo Guillén, J. I. El poblado y necrópolis prehistóricos de 'Riols I', Mequinenza, Zaragoza. Campaña de urgencia. *Arqueología Aragonesa 1985* **1985**, 31–35 (1987).
4. Royo Guillén, J. I. Las necrópolis de los campos de urnas el valle medio del Ebro, como precedente del mundo funerario celtibérico. in *Necrópolis celtibéricas: II Simposio sobre los celtíberos [celebrado en Daroca (Zaragoza), del 28 al 30 de abril de 1988]* 123–136 (Institución' Fernando el Católico', 1990).
5. Royo Guillén, J. I. Estudio de materiales de Los Castellets de Mequinenza. Campaña de 1990. *Arqueología Aragonesa 1990* **1990**, 81–87 (1992).
6. Royo Guillén, J. I. Ritual funerario y cultura material en las necrópolis tumulares de Los Castellets de Mequinenza (Zaragoza): Una aportación al estudio del Bronce Final/Hierro I en el N. E. peninsular. in *Models d´ocupació, transformació i explotació del territori entre el 1600 i el 500 a.n.e. a la Catalunya Meridional i zones limítrofes de la depresió de l´Ebre.* (Edition: Gala, Revista d´Arqueologia, Antropologia i Patrimoni, 3-5. Museu Nacional de Sant Feliu de Codines (Catalunya, España), 1996).
7. Royo Guillén, J. I. Tipología funeraria, ritos y ofrendas en las necrópolis del valle del Ebro durante la Primera Edad del Hierro (s. VIII-s. V a. C.). in 41–58 (2000).
8. Royo Guillén, J. I., García Martínez de Lagrán, I. & Tejedor Rodríguez, C. La aplicación de la Estadística Bayesiana en la periodización de la I^{a} Edad del

Hierro en el Valle Medio del Ebro. in (dialnet.unirioja.es, 2018).

9. Beltrán LLoris, M. *Museo de Zaragoza : Secciones de Arqueología Y Bellas Artes*. vol. 101 (1976).
10. Cuadrado Díaz, E. La Almoloya, nuevo poblado de la cultura de El Argar. *Anales de la Universidad de Murcia.* (1945).
11. Lorenzo Lizalde, J. I. Paleoantropología de la necrópolis Bronce Final - Campos de Urnas, de Los Castellets II de Mequinenza (Zaragoza). in *Arqueología Aragonesa 1988-89* (ed. de Cultura y Educación, D.) vol. Arqueología Aragonesa 1988-89 447–451 (1991).
12. Royo Guillén, J. I. & Pérez i Conill, J. Los vasos bitroncocónicos de las necrópolis de Los Castellets y Can Missert y los primeros campos de túmulos y urnas en el noreste peninsular. *Bolskan* **27**, 55–86 (2019).
13. Gisbert, M. & Pastor, M. *Guia de Cuevas Y Simas de La Provincia de Zaragoza*. (2009).
14. Laborda-Lorente, R., Villalba-Mouco, V. & Gisbert-Leon, M. La Cueva de los Piojos (Ricla, Zaragoza). Nuevos datos sobre el uso de las cuevas como recintos funerarios en las estribaciones septentrionales del Sistema Ibérico. in (2017).
