# Supplementary material for: Genomic insights from a final Bronze Age community buried in a collective tumulus in an Urnfield settlement in Northeastern Iberia
Source: Commun Biol. 2025 Aug 28;8:1299. doi: 10.1038/s42003-025-08668-7 (PMC12394608; doi:10.1038/s42003-025-08668-7)
Supplement: Supplementary file 2 — Description of Additional Supplementary files [file 42003_2025_8668_MOESM2_ESM.pdf]

Description of Additional Supplementary files

File name: Supplementary Data 1 and 2

Description: Numerical source data for graphs and charts can be found in Supplementary Data 1 and 2.
