# Supplementary material for: Genomic insights from a final Bronze Age community buried in a collective tumulus in an Urnfield settlement in Northeastern Iberia
Source: Commun Biol. 2025 Aug 28;8:1299. doi: 10.1038/s42003-025-08668-7 (PMC12394608; doi:10.1038/s42003-025-08668-7)
Supplement: Supplementary file 5 — Reporting summary [file 42003_2025_8668_MOESM5_ESM.pdf]

nature portfolio

Corresponding author(s): Vanessa Villalba-Mouco
Wolfgang Haak

Last updated by author(s): August 2nd, 2025

Reporting Summary

Nature Portfolio wishes to improve the reproducibility of the work that we publish. This form provides structure for consistency and transparency in reporting. For further information on Nature Portfolio policies, see our Editorial Policies and the Editorial Policy Checklist.

Statistics

For all statistical analyses, confirm that the following items are present in the figure legend, table legend, main text, or Methods section.

n/a	Confirmed	
☐	☒	The exact sample size (*n*) for each experimental group/condition, given as a discrete number and unit of measurement
☐	☒	A statement on whether measurements were taken from distinct samples or whether the same sample was measured repeatedly
☐	☒	The statistical test(s) used AND whether they are one- or two-sided<br>*Only common tests should be described solely by name; describe more complex techniques in the Methods section.*
☐	☒	A description of all covariates tested
☐	☒	A description of any assumptions or corrections, such as tests of normality and adjustment for multiple comparisons
☐	☒	A full description of the statistical parameters including central tendency (e.g. means) or other basic estimates (e.g. regression coefficient) AND variation (e.g. standard deviation) or associated estimates of uncertainty (e.g. confidence intervals)
☐	☒	For null hypothesis testing, the test statistic (e.g. *F*, *t*, *r*) with confidence intervals, effect sizes, degrees of freedom and *P* value noted<br>*Give P values as exact values whenever suitable.*
☒	☐	For Bayesian analysis, information on the choice of priors and Markov chain Monte Carlo settings
☒	☐	For hierarchical and complex designs, identification of the appropriate level for tests and full reporting of outcomes
☒	☐	Estimates of effect sizes (e.g. Cohen's *d*, Pearson's *r*), indicating how they were calculated

Our web collection on statistics for biologists contains articles on many of the points above.

Software and code

Policy information about availability of computer code

Data collection	Oxcal 4.4<br>nf-core/eager 2 pipeline v.2.3.5<br>AdapterRemoval v2<br>BWA v.0.7.12<br>DeDup v.0.12.1<br>mapDamage v.2.0.9<br>samtools (v1.3)<br>pileupCaller v8.6.5
Data analysis	ANGSD (0.910)<br>ContamMix<br>ADMIXTOOLS (5.1) (pq3Pop, qpDstats, qpGraph, qpF4Ratio)<br>Haplogrep 3<br>R version 4.2.3<br>haproh 0.64<br>ancIBD v0.6<br>BREADR<br>python3<br>KIN<br>EIGENSOFT (v6.0.1)

GLIMPSE

For manuscripts utilizing custom algorithms or software that are central to the research but not yet described in published literature, software must be made available to editors and reviewers. We strongly encourage code deposition in a community repository (e.g. GitHub). See the Nature Portfolio guidelines for submitting code & software for further information.

Data

Policy information about availability of data

All manuscripts must include a data availability statement. This statement should provide the following information, where applicable:
- Accession codes, unique identifiers, or web links for publicly available datasets
- A description of any restrictions on data availability
- For clinical datasets or third party data, please ensure that the statement adheres to our policy

Sequenced data in FASTQ and BAM format is accessible through the European Nucleotide Archive (ENA) under the project name PRJEB85840. All other data are available from the corresponding author upon reasonable request.

Research involving human participants, their data, or biological material

Policy information about studies with human participants or human data. See also policy information about sex, gender (identity/presentation), and sexual orientation and race, ethnicity and racism.

Reporting on sex and gender	*Use the terms sex (biological attribute) and gender (shaped by social and cultural circumstances) carefully in order to avoid confusing both terms. Indicate if findings apply to only one sex or gender; describe whether sex and gender were considered in study design; whether sex and/or gender was determined based on self-reporting or assigned and methods used. Provide in the source data disaggregated sex and gender data, where this information has been collected, and if consent has been obtained for sharing of individual-level data; provide overall numbers in this Reporting Summary. Please state if this information has not been collected. Report sex- and gender-based analyses where performed, justify reasons for lack of sex- and gender-based analysis.*
Reporting on race, ethnicity, or other socially relevant groupings	*Please specify the socially constructed or socially relevant categorization variable(s) used in your manuscript and explain why they were used. Please note that such variables should not be used as proxies for other socially constructed/relevant variables (for example, race or ethnicity should not be used as a proxy for socioeconomic status). Provide clear definitions of the relevant terms used, how they were provided (by the participants/respondents, the researchers, or third parties), and the method(s) used to classify people into the different categories (e.g. self-report, census or administrative data, social media data, etc.) Please provide details about how you controlled for confounding variables in your analyses.*
Population characteristics	*Describe the covariate-relevant population characteristics of the human research participants (e.g. age, genotypic information, past and current diagnosis and treatment categories). If you filled out the behavioural & social sciences study design questions and have nothing to add here, write "See above."*
Recruitment	*Describe how participants were recruited. Outline any potential self-selection bias or other biases that may be present and how these are likely to impact results.*
Ethics oversight	*Identify the organization(s) that approved the study protocol.*

Note that full information on the approval of the study protocol must also be provided in the manuscript.

Field-specific reporting

Please select the one below that is the best fit for your research. If you are not sure, read the appropriate sections before making your selection.

☐ Life sciences ☐ Behavioural & social sciences ☒ Ecological, evolutionary & environmental sciences

For a reference copy of the document with all sections, see nature.com/documents/nr-reporting-summary-flat.pdf

Ecological, evolutionary & environmental sciences study design

All studies must disclose on these points even when the disclosure is negative.

Study description	We analyse ancient genomic data from two archaeological sites on the Northeastern Iberia, the Final Bronze Age site of Los Castellets II, and the Iron Age site of Cueva de los Piojos. We performed a population genomic study as well as biological kinship of the humans individuals.
Research sample	We studied the archaeological remains of individuals corresponding the Los Castellets II and Cueva de los Piojos sites
Sampling strategy	All samples from which ancient DNA data were generated are reported.

Data collection	Data collection for ancient DNA sequence data was made through the sequencing of DNA extracted from petrous bones or teeth; undertaken in dedicated clean lab facilities at the Max Planck Institute for Evolutionary Anthropology (MPI-EVA).
Timing and spatial scale	We study samples from two archaeological sites on the Northeastern Iberia, the Final Bronze Age site of Los Castellets II, and the Iron Age site of Cueva de los Piojos
Data exclusions	No ancient DNA data were excluded where available for the sites and phases described.
Reproducibility	No experimental findings are reported.
Randomization	N/A
Blinding	N/A

Did the study involve field work? ☐ Yes ☒ No

Reporting for specific materials, systems and methods

We require information from authors about some types of materials, experimental systems and methods used in many studies. Here, indicate whether each material, system or method listed is relevant to your study. If you are not sure if a list item applies to your research, read the appropriate section before selecting a response.

Materials & experimental systems

n/a	Involved in the study	
☒	☐	Antibodies
☒	☐	Eukaryotic cell lines
☐	☒	Palaeontology and archaeology
☒	☐	Animals and other organisms
☒	☐	Clinical data
☒	☐	Dual use research of concern
☒	☐	Plants

Methods

n/a	Involved in the study	
☒	☐	ChIP-seq
☒	☐	Flow cytometry
☒	☐	MRI-based neuroimaging

Palaeontology and Archaeology

Specimen provenance	All individuals analysed in this study were obtained from the deposits of the Museum of Zaragoza with permission from the Cultural Heritage Department of Gobierno de Aragón. The archaeological sampling permit was issued on July 5th, 2019, by Ignacio Escuáin Borao, General Director of Cultural Heritage at the Gobierno de Aragón, and can be consulted in Supplementary Information 4.
Specimen deposition	The specimens have been returned to the storage facilities of the Museum of Zaragoza. The original archaeological codes are available in Supplementary Data 1.1, as well as with the archaeological materials, for consultation or replication analyses if needed.
Dating methods	Four samples out of 33 from the same collective tumulus form Los Castellets II were radiocarbon dated, as well as the single individual analysed from Los Piojos cave. Radiocarbon dating was carried out from ultra-filtrated collagen and using accelerated mass spectrometry (AMS) at the Curt-Engelhorn-Zentrum Archäometrie gGmbH in Mannheim, Germany (raw and calibrated dates are available in Supplementary Data 1.1). All samples were calibrated using the IntCal20 calibration curve with Oxcal 4.4 62. All 14C dates in this study are consistent with the archaeological chronology based in stratigraphy and grave goods.

☒ Tick this box to confirm that the raw and calibrated dates are available in the paper or in Supplementary Information.

Ethics oversight	All necessary permits were obtained from the relevant institutions (see Supplementary Information 4), and analyses were conducted with respect for the archaeological context and in compliance with established ethical guidelines for the study of ancient DNA. The archaeological researchers, supported by the Cultural Heritage Department of the Gobierno de Aragón, approved and provided guidance on the sample selection and sampling protocols (see Supplementary Information 4).

Note that full information on the approval of the study protocol must also be provided in the manuscript.

Plants

Seed stocks	*Report on the source of all seed stocks or other plant material used. If applicable, state the seed stock centre and catalogue number. If plant specimens were collected from the field, describe the collection location, date and sampling procedures.*
Novel plant genotypes	*Describe the methods by which all novel plant genotypes were produced. This includes those generated by transgenic approaches, gene editing, chemical/radiation-based mutagenesis and hybridization. For transgenic lines, describe the transformation method, the number of independent lines analyzed and the generation upon which experiments were performed. For gene-edited lines, describe the editor used, the endogenous sequence targeted for editing, the targeting guide RNA sequence (if applicable) and how the editor was applied.*
Authentication	*Describe any authentication procedures for each seed stock used or novel genotype generated. Describe any experiments used to assess the effect of a mutation and, where applicable, how potential secondary effects (e.g. second site T-DNA insertions, mosiacism, off-target gene editing) were examined.*
